# The Micropillar Structure on Silk Fibroin Film Influence Intercellular Connection Mediated by Nanotubular Structures

**DOI:** 10.3390/ma7064628

**Published:** 2014-06-18

**Authors:** Renchuan You, Xiufang Li, Yamei Xu, Yu Liu, Shenzhou Lu, Mingzhong Li

**Affiliations:** National Engineering Laboratory for Modern Silk, College of Textile and Clothing Engineering, Soochow University, No. 199 Ren’ai Road, Industrial Park, Suzhou 215123, Jiangsu, China; E-Mails: 20124015002@suda.edu.cn (R.Y.); 20114215026@suda.edu.cn (X.L.); 20124215007@suda.edu.cn (Y.X.); liuyu@suda.edu.cn (Y.L.); lushenzhou@suda.edu.cn (S.L.)

**Keywords:** micropillars, silk fibroin, tunneling nanotubes, intercellular connection

## Abstract

Tunneling nanotubes are important membrane channels for cell-to-cell communication. In this study, we investigated the effect of the microenvironment on nanotubular structures by preparing a three-dimensional silk fibroin micropillar structure. In previous reports, tunneling nanotubes were described as stretched membrane channels between interconnected cells at their nearest distance. They hover freely in the cell culture medium and do not contact with the substratum. Interestingly, the micropillars could provide supporting points for nanotubular connection on silk fibroin films, where nanotubular structure formed a stable anchor at contact points. Consequently, the extension direction of nanotubular structure was affected by the micropillar topography. This result suggests that the hovering tunneling nanotubes in the culture medium will come into contact with the raised roadblock on the substrates during long-distance extension. These findings imply that the surface microtopography of biomaterials have an important influence on cell communication mediated by tunneling nanotubes.

## 1. Introduction

Cells communicate with each other via a variety of complex intercellular interactions, some of which occur through gap junctions [1] and synapses [2]. An emerging mechanism of cell communication has been proposed since the 2004 report of rat pheochromocytoma PC12 cells that were bridged by tunneling nanotubes (TNTs), known as membrane nanotubes [3]. Subsequently, the nanotubular structures have been found in various types of cells such as fibroblasts [4], cardiac myocytes [4,5], epithelial cells [5,6], immune cells [7,8,9], primary neurons [10] and astrocytes [10,11]. The nanotubular channels allow the exchange of components between animal cells [12]. Moreover, increasing evidence has shown that nanotubular channels provided backdoors for the intercellular spread of pathogens [8,13,14].

TNTs were initially described as thin intercellular membrane channels with diameters ranging from 50 to 200 nm, displaying lengths of up to several cell diameters [3]. TNT displayed a pronounced sensitivity to light excitation, mechanical stress and chemical fixation, leading to the rupture of many TNTs [3]. So far, similar structures with various length and diameter have been reported in different cell lines [12]. Furthermore, a large number of researches have been performed to investigate the formation mechanism, cargo and signal transfer process of nanotubular structure [15,16,17,18,19,20,21]. TNT may connect to a neighboring cell by anchoring junctions in human tumor urothelial cells (T24 cell), and the anchoring proteins are N-cadherin and its adaptor protein β-catenin [19]. Moreover, nanoparticles could be transported in the membrane nanotubes of cardiac myocytes and neuronal cells [20,21]. However, most of the present research is focused on the morphology of nanotubes and transport of cellular components, none of these studies examine the effect of microenvironment on the TNTs. Interestingly, TNTs hover freely in the cell culture medium and do not contact with the substratum, and they were stretched between interconnected cells attached at their nearest distance [3]. 3D analysis further showed that nanotubular structure between cells at their nearest distance has no contact to the substrate and are aligned mostly parallel to the surface [22]. These results demonstrated that TNTs can span a long distance by hovering extension and do not contact with substrate in the flat culture system. However, topographical structures at the micro to nanometer scales have been shown to affect cell behavior, including morphological changes and functional alterations [23,24,25,26]. In particular, surface topography showed significant effect on the extension and morphology of short membrane protrusion, such as filopodia and lamellipodia [23,27]. TNT is a longer membrane protrusion connecting surrounding cells over long distances, which has a similar morphology and diameter with filopodia [28]. Therefore, we proposed that surface topography may change the extension of TNTs. Although TNTs hover freely in the culture medium on the flat surface, the extension might be disturbed by surface microstructure, leading to a potential influence on cell communication. It was therefore of interest to study whether a micropillar topography affect the extension of hovering nanotubular structure.

Silk fibroin (SF) produced by silkworms was studied as a promising biomaterial due to its aqueous processability, biocompatibility, and biodegradability [29,30,31]. In the present study, we prepared a micropillar structure on SF films to study the effect of microtopography on nanotubular connection. We found that the SF micropillars provided supporting points for nanotubular connection between bone marrow derived mesenchymal stem cells (BMSCs), and further influenced the extension direction of nanotubular structure.

## 2. Results and Discussion

### 2.1. Surface Characterization of Silk Fibroin Films

**Figure 1:**
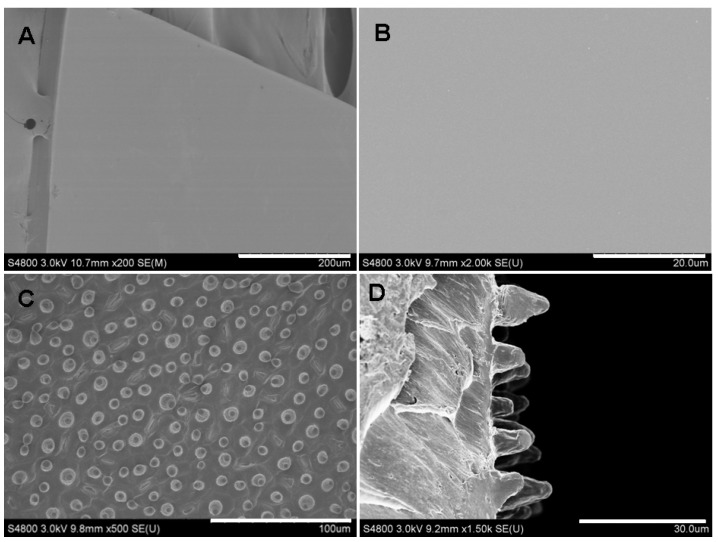
Surface characterization of silk fibroin (SF) films: (**A**) flat film; (**B**) magnified image of flat film surface; (**C**) the surface of microstructured film and (**D**) magnified cross-section view of the micropillar structure. Scale bars: (**A**) 200 μm; (**B**) 20 μm; (**C**) 100 μm; (**D**) 30 μm.

SF is a natural fibrous protein that has been widely used as a biomaterial because of its excellent biocompatibility and biodegradability [29]. Furthermore, Silk fibroin can be regenerated into aqueous solution, which is advantageous for the preparation of the biomaterials with various microstructures [23,32]. Scanning electron microscopy (SEM) images of the surface topography of the SF films showed that the SF film prepared on a polystyrene dish was flat (Figure 1A,B). A micropillar structure was successfully obtained from the Poly(dimethylsiloxane) (PDMS) mold (Figure 1C), and the space between adjacent micropillars mainly ranged from 5 to 20 μm. Natural lotus leaf surfaces are composed of micro/nano binary cooperative structure [33]. The nanoscale structure will be destroyed after PDMS introduction, thus only the microscale topography was replicated. The magnified cross-section (Figure 1D) shows the diameter and height of the micropillars (5–10 μm and 5–15 μm, respectively).

### 2.2. The Nanotubular Connection of BMSCs on the Silk Fibroin Films

Since TNTs was firstly reported in PC12 cells, the intercellular membrane channels have been discovered in various cell types. TNTs establish intercellular tubular membrane channels for the exchange of cell surface molecules and cytoplasmic contents such as calcium ions, plasma membrane components, endosome-related organelles and mitochondria [3,4,5,6,12,13,14,15,16,17]. In addition to the intercellular exchange of small molecules and organelles, the bidirectional spread of electrical signals between TNT-connected cells has been demonstrated [15,16]. The membrane channels have been shown to be involved in the intercellular spread of pathogens and transfer of aberrant proteins responsible for disease, including bacteria, viruses and prions and misfolded huntingtin [28]. Moreover, the intercellular connection was also observed in coculture systems [4,5], and the connection between different cell types may contribute to the pathophysiology of diseases such as cardiac fibrosis [4]. These results demonstrated that the nanotubular bridges showed important functions in cell-to-cell communication and the pathophysiology of some diseases. Furthermore, the recent reports showed that M-Sec (Tumor necrosis factor induced protein 2; TNFaip2) is a key molecule for TNT formation. M-Sec can induce the formation of functional TNTs through interaction with the Ral-exocyst pathway, and the remodeling of the actin cytoskeleton and vesicle trafficking are involved in M-Sec-mediated TNT formation [17,18]. These discoveries expanded the knowledge regarding the mechanisms of TNT-mediated cell communication. However, these researches are focused on the morphology and intercellular transport of membrane nanotubes, whereas the effect of microenvironment on nanotubular connection has not been investigated.

Cell-material interaction is a key fundamental topic in the fields of biomaterials and regenerative medicine. Microscale topography on material surface influences adhesion, morphology, migration and differentiation in a wide variety of cell types, ranging from fibroblasts to MSCs [25]. The geometry and size of substrates can influence the extension and contact of the protrusions from cell membrane, such as filopodia and lamellipodia, then further results in the changes of cell morphology and functions [23,27]. Therefore, we proposed that surface topography may influence the extension of TNTs. TNT is a thin dynamic filopodia-like membrane protrusion connecting surrounding cells over long distances. Different from the structure of filopodia, TNTs have been described as stretched membrane channels between interconnected cells at their nearest distance, meanwhile they hover freely in the cell culture medium and do not contact with the substratum on flat surface [3,22]. However, the extension and connection of TNTs on microstructured surface is unclear. In order to study the effect of microenvironment on the nanotubular connection, we established a microstructured SF film to investigate the influence of micropillar structure on the intercellular nanotubular connection.

**Figure 2:**
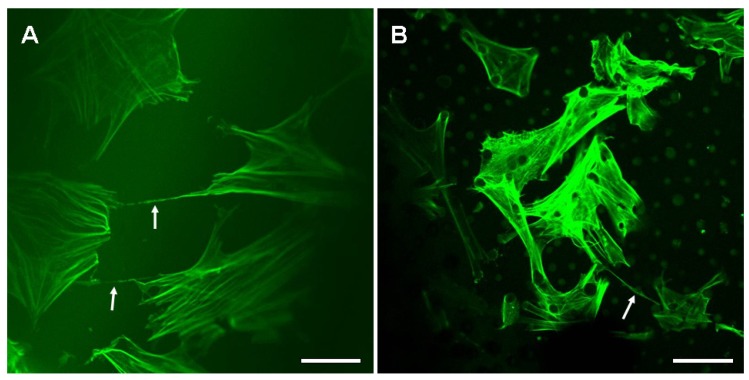
Confocal laser scanning microscopy (CLSM) images of nanotubular connection on the SF films: (**A**) cells on a flat film; (**B**) cells on a microstructured film. Scale bars: 50 μm.

**Figure 3:**
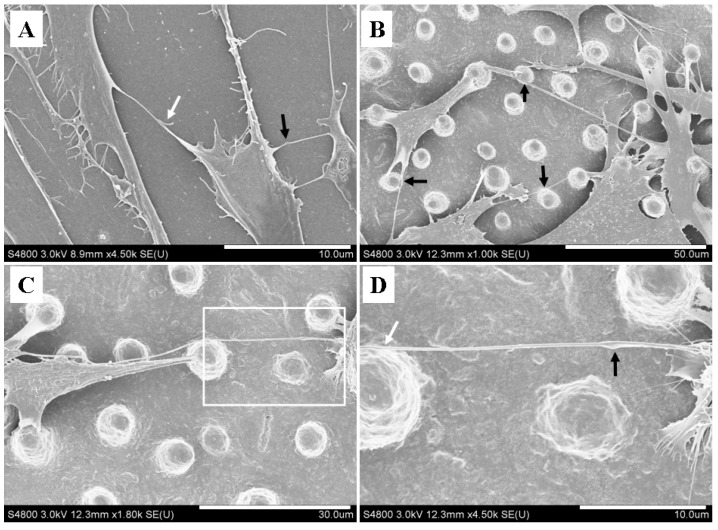
SEM images of nanotubular network on the SF films: (**A**) cells on a flat film; (**B**,**C**) cells on microstructured films and (**D**) magnified image of the boxed region from Figure 3C. Scale bars: (**A**,**D**) 10 μm; (**B**) 50 μm; (**C**) 30 μm.

TNT structures contain F-actin, and the nanotubular structure formation is generated by actin-driven protrusions of the cytoplasmic membrane [3,34]. After cell culture for 3 days, the F-actin labeled fluorescence images showed the formation of nanotubular connection between neighboring BMSCs on SF films (Figure 2, white arrows). On the flat film, the cells randomly spread and protruded plasma membranes, and protruded stretched nanotubes to connect surrounding cells (Figure 2A). On the microstructured surface, the SF micropillar structure provided a three-dimensional (3D) microenvironment for cell growth. Cell morphologies were regulated by the distribution of local micropillars. The cells spread on the top of the micropillars, and the cell membranes wrapped around the micropillars (Figure 3B). Several nanotubular structures, whose lengths ranged from several to tens of microns, could protrude from individual cell to form a nanotubular network with the surrounding cells (Figure 3A,B). The nanotubular structure provided a seamless connection between cells (Figure 3C). TNTs may connect to each other through two mechanisms: actin-driven filopodia-like protrusions of donor cells can connect to an acceptor cell, or attached cells can detach from one another [6,35,36]. As shown in Figure 3A, two filopodia-like protrusions were converging each other, might implying that the protrusions from individual cell were fusing into a nanotubular structure to connect neighboring cells. Furthermore, previous studies have demonstrated that large organelles and vesicles can be transported through TNT [35,37]. The focal bulges were observed along the lengths of some nanotubes (Figure 3A,D, black arrows), which is consistent with Wittig *et al*.’s observation [38], indicating a possible transport of organelles or vesicles along nanotubes.

### 2.3. Micropillars Provided Supporting Points for Nanotubular Connection

**Figure 4:**
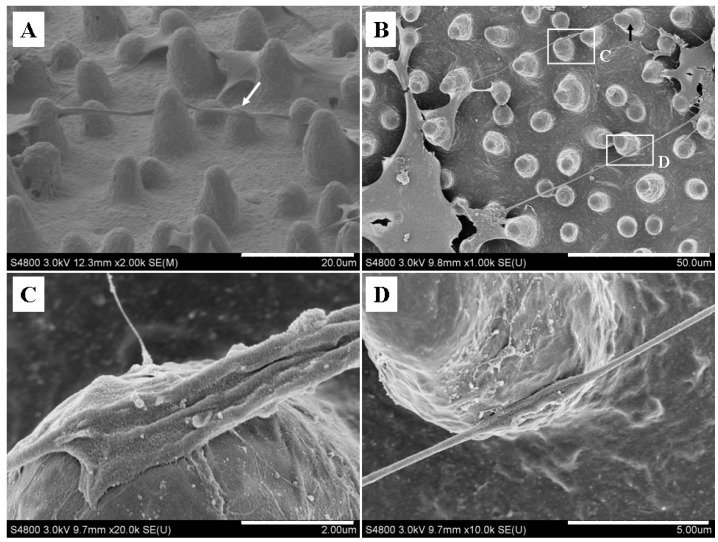
SF micropillars provided supporting points for nanotubular connection. (**A**) Tunneling nanotubes (TNT) extended on the top of micropillars; (**B**) nanotubular structures between neighboring cells; (**C**,**D**) represent magnifications of the boxed regions in Figure 4B. Scale bars: (**A**) 20 μm; (**B**) 50 μm; (**C**) 2 μm; (**D**) 5 μm.

In contrast to other cellular protrusions, TNTs hover freely in the cell culture medium and do not contact with the substratum in the standard culture system [35]. Furthermore, 3D analysis revealed that nanotubular structures between cells connected at their nearest distance without contact with substrate and were aligned mostly parallel to the substrate surface [22]. The results confirmed that TNTs can span a long distance by hovering in the culture medium without contact with substrate. Here, the nanotubular structures were stretched between interconnected cells on the flat SF films (Figure 2A and Figure 3A), which were similar to in the standard cell culture system. On the microstructured SF films, the nanotubular structure extended on the top of micropillars without contact with the underlaying substrate (Figure 4A); however, these hovering nanotubular structures contacted with micropillars and extended along the surface of micropillar (Figure 4A, white arrow). The result implied that the micropillars on the SF film may have a significant effect on nanotubular connection of BMSCs.

To further determine the influence of the micropillar structure on the nanotubular connection, we observed the contact points between the nanotube and micropillar (Figure 4B). At the contact points, a sheet and a lump-like structure formed at the micropillar surface (Figure 4C,D). The sheet-like structure wrapped around the micropillars to form a stable anchor. Confocal fluorescence imaging (Figure 2B, white arrow) confirmed the wrapped anchoring at the micropillar surface. These results indicated that the SF micropillars provided supporting points for nanotubular connection of BMSCs. For the formation of an intercellular connection, membrane nanotube may begin growing as filopodium-like pertrusion [19], then extend and slide to connect target cell in culture medium at a hovering form. On the flat film, TNT can form a stretched connection. However, the raised micropillars would disturb and prevent the extension of long nanotubes. Consequently, when the ongoing nanotube encounter a micropillar during long-distance extension, the nanotube can interact with micropillar and form a stable anchoring at contact points for continued extension. The results impliy TNTs can form a stable anchoring for further extension when encounter a raised roadblock.

### 2.4. Micropillars Influenced the Extension Direction of Nanotubular Structure

**Figure 5:**
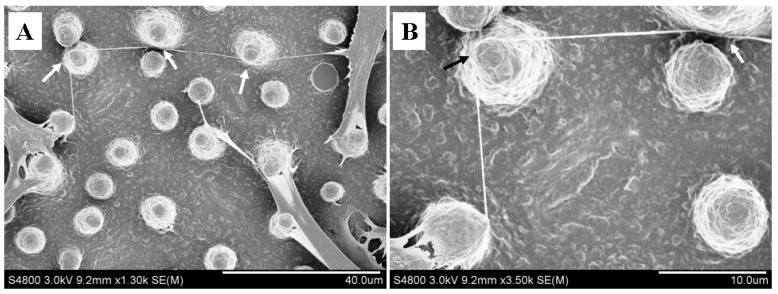
The extension direction of nanotubular connection was influenced by micropillar structure. (**A**) A circuitous extension between interconnected cells; (**B**) magnified image of swerved section in Figure 5A. Scale bars: (**A**) 40 μm; (**B**) 10 μm.

The 3D micropillar structure provided a spatially structured microenvironment for the hovering extension of nanotubular structure, and the supporting points might give rise to the change of extension route. As shown in Figure 4B, the micropillar (black arrow) influenced the extension direction of nanotubular structure. However, the nanotubular structure was described as stretched connection between cells at their nearest distance in standard culture [3,12]. Therefore, we further observed the extension direction of nanotubular structure on microstructured films. Interestingly, the nanotubular structure was able to bridge neighboring cells in a circuitous route rather than at the nearest distance (Figure 5A). Several micropillars provided supporting points for the circuitous extension (Figure 5A, white arrows), and the nanotubular structure was stretched between adjacent micropillars. At the micropillar surface, the nanotubular structure sharply swerved toward target cell (Figure 5B, black arrow), indicating that the micropillar topography affected the extension direction of nanotubular structure through stable supporting points. These findings demonstrated that a micropillar array provided supporting points for nanotubular structure and further affected their extension direction, implying that surface microtopography significantly affects intercellular nanotubular connection.

Cell-to-cell communication plays a crucial role in the development and maintenance of multicellular organisms [19]. TNTs have been shown to transport various cellular components, including cytoplasmic contents, plasma membrane components and organelles [35]. On the microstructured surface, membrane nanotubes can interact with micropillar structure and showed a circuitous extension, suggesting surface topography may have an important effect on the intercellular transport and communication. To better understand the effect of microstructure on the cell-to-cell communication mediated by nanotubular structures, the change of intercellular transport should be further observed in the future. On the other hand, TNTs are brittle to light excitation and mechanical stress, and TNTs showed dynamic nature and transient lifetime [3]. Moreover, no TNT-specific protein markers are known [28]. These factors make it difficult to clarify the molecular mechanism of TNT-micropillar interaction. Future research on TNTs should pay attention to the molecular mechanism of TNT-substrate interaction at anchoring points, which will facilitate the clarification of the sensing mechanism of TNT to substrate topography.

## 3. Materials and Methods

### 3.1. Preparation of Silk Fibroin Solution

Regenerated SF solution was prepared as described previously [23]. Briefly, *Bombyx mori* raw silk fibers (Huzhou, China) were degummed three times in 0.05 wt% Na_2_CO_3_ at 98–100 °C for 30 min and dried in an oven after rinsing. The degummed fibroin was dissolved in a ternary CaCl_2_:CH_3_CH_2_OH:H_2_O solvent (mole ratio, 1:2:8) at 72 ± 2 °C for 1 h. A 3.2 wt% SF solution was obtained after dialysis (MWCO 9000–14000) in deionized water for 4 days.

### 3.2. Mold Preparation

PDMS (Sylgard 184, Dow Corning, Midland, MI, USA) was mixed at a weight ratio of 10:1 (base:agent), degassed under vacuum to remove bubbles and then poured into the upper side of fresh lotus leaves. The PDMS was heated at 40 °C for 8 h and peeled from the lotus leaves. The solidified PDMS was cut into 2.5 cm × 2.5 cm squares after thorough rinsing.

### 3.3. Preparation of Silk Fibroin Films

The SF solution was centrifuged at 5000 r/min for 10 min to remove particles and bubbles, and 1 mL of a 3% SF solution was then cast on the PDMS molds. The molds were covered with a venting lid and allowed to air dry at 25 °C in a clean environment [32]. Flat SF films were obtained by casting the SF solution on polystyrene dishes, and the film thickness was regulated by adjusting the ratio between the SF solution and the mold area. The films were peeled from the molds after drying and then immersed in 80% ethanol for 2 h to create water-insoluble SF films through inducing structural transition from random coil to the β-sheet [29].

### 3.4. Cell Culture

Bone marrow was flushed from the femur of 4-6-week-old Sprague-Dawley rats (SPF grade, male) and resuspended in DMEM/F-12 culture medium (HyClone, Logan, UT, USA) supplemented with 10% fetal bovine serum (FBS; HyClone, Logan, UT, USA) and a 1% penicillin-streptomycin solution (Beyotime, Nantong, China). The animal experiments were approved by the Jiangsu Province in experimental animals management rules ([2008] No. 26) [23]. The cells were cultured in a humidified incubator with 5% CO_2_ at 37 °C, and fresh culture medium was added to remove nonadherent cells after 24 h. The culture medium was replaced every 3 days, and the cells were passaged when they were almost confluent. To prevent the loss of stem cell characteristics in long-term *in vitro* culturing, only BMSCs from passages 3–6 were used for experiments.

The SF films were cut into discs of 1.5 cm diameter and immersed in 75% ethanol for 15 min. Sterilized samples were then placed in 24-well plates and washed three times with sterile phosphate-buffered saline (PBS; 0.1 M, pH 7.4) for cell seeding. The samples were fixed on the bottom of the culture well using sterilized polystyrene tubes, and BMSCs were then seeded on the SF films at 4 × 10^4^ cells/cm^2^. Cells were fixed for observation after 3 days culture.

### 3.5. Scanning Electron Microscopy

SEM (S-4800, Hitachi, Tokyo, Japan) was used to observe the surface morphology of the SF films. The cell culture samples were rinsed in PBS three times and fixed in 2.5% glutaraldehyde at 4 °C for 6 h followed by three washes in PBS. The fixed samples were dehydrated by an ascending graded ethanol series (50%, 70%, 90% and 99.7%) for 5 min each step and then further dried using hexamethyldisiloxane (HMDS; Sigma-Aldrich, St. Louis, MO, USA) for 3 min. The dried samples were observed using SEM after gold sputter coating.

### 3.6. Immunofluorescence Labeling

BMSCs were rinsed with PBS and fixed with 4% paraformaldehyde in PBS for 15 min at room temperature, permeabilized with 0.2% Triton X-100 in PBS for 5 min and then blocked with 2% BSA (Millipore, Bedford, MA, USA) in PBS for 30 min. The cells were incubated in 5 μg/mL FITC-phalloidin (Sigma-Aldrich, St. Louis., MO, USA) for 2 h at room temperature, and the samples were then rinsed three times with PBS for confocal laser scanning microscopy (CLSM; IX81/FV1000, Olympus, Tokyo, Japan) observation by applying a laser beam at a wavelength of 488 nm.

## 4. Conclusions

Here we provide the first evidence on the effect of biomaterial surface microtopography on the nanotubular connection of BMSCs. We found that nanotubular structure adapts to the 3D micropillar topography. The spatially arranged micropillars on the SF film provided supporting points for a nanotubular structure. Furthermore, the extension direction of nanotubular connection is affected by the micropillar structure. These findings show the crucial effect of the surface microtopography on the intercellular connection mediated by nanotubular structure.

## References

[1] Maeda S., Tsukihara T. (2011). Structure of the gap junction channel and its implications for its biological functions. Cell. Mol. Life Sci..

[2] Fields R.D., Stevens-Graham B. (2002). New insights into neuron-glia communication. Science.

[3] Rustom A., Saffrich R., Markovic I., Walther P., Gerdes H.H. (2004). Nanotubular highways for intercellular organelle transport. Science.

[4] He K.M., Shi X.L., Zhang X.J., Dang S., Ma X.W., Liu F., Xu M., Lv Z., Han D., Fang X. (2011). Long-distance intercellular connectivity between cardiomyocytes and cardiofibroblasts mediated by membrane nanotubes. Cardiovasc. Res..

[5] Koyanagi M., Brandes R.P., Haendeler J., Zeiher A.M., Dimmeler S. (2005). Cell-to-cell connection of endothelial progenitor cells with cardiac myocytes by nanotubes: A novel mechanism for cell fate changes?. Circ. Res..

[6] Ferrati S., Shamsudeen S., Summers H., Rees P., Abbey J.V.A., Schmulen J., Liu X., Wong S.T.C., Bean A.J., Ferrari M. (2012). Inter-endothelial transport of microvectors using cellular shuttles and tunneling nanotubes. Small.

[7] Önfelt B., Nedvetzki S., Yanagi K., Davis D.M. (2004). Cutting edge: Membrane nanotubes connect immune cells. J. Immunol..

[8] Sowinski S., Jolly C., Berninghausen O., Purbhoo M.A., Chauveau A., Köhler K., Oddos S., Eissmann P., Brodsky F.M., Hopkins C. (2008). Membrane nanotubes physically connect T cells over long distances presenting a novel route for HIV-1 transmission. Nat. Cell Biol..

[9] Chauveau A., Aucher A., Eissmann P., Vivier E., Davis D.M. (2010). Membrane nanotubes facilitate long-distance interactions between natural killer cells and target cells. Proc. Natl. Acad. Sci. USA.

[10] Wang Y., Cui J., Sun X., Zhang Y. (2011). Tunneling-nanotube development in astrocytes depends on p53 activation. Cell Death Differ..

[11] Zhu D.H., Tan K.S., Zhang X.L., Sun A.Y., Sun G.Y., Lee J.C.M. (2005). Hydrogen peroxide alters membrane and cytoskeleton properties and increases intercellular connections in astrocytes. J. Cell Sci..

[12] Gerdes H.H., Bukoreshtliev N.V., Barroso J.F.V. (2007). Tunneling nanotubes: A new route for the exchange of components between animal cells. FEBS Lett..

[13] Önfelt B., Nedvetzki S., Benninger R.K.P., Purbhoo M.A., Sowinski S., Hume A.N., Seabra M.C., Neil M.A.A., French P.M.W., Davis D.M. (2006). Structurally distinct membrane nanotubes between human macrophages support long-distance vesicular traffic or surfing of bacteria. J. Immunol..

[14] Gousset K., Schiff E., Langevin C., Marijanovic Z., Caputo A., Browman D.T., Chenouard N., Chaumont F., Martino A., Enninga J. (2009). Prions hijack tunneling nanotubes for intercellular spread. Nat. Cell Biol..

[15] Pascoal P., Kosanic D., Gjoni M., Vogel H. (2010). Membrane nanotubes drawn by optical tweezers transmit electrical signals between mammalian cells over long distances. Lab Chip.

[16] Wang X., Veruki M.L., Bukoreshtliev N.V., Hartveit E., Gerdes H.H. (2010). Animal cells connected by nanotubes can be electrically coupled through interposed gap-junction channels. Proc. Natl. Acad. Sci. USA.

[17] Hase K., Kimura S., Takatsu H., Ohmae M., Kawano S., Kitamura H., Ito M., Watarai H., Hazelett C.C., Yeaman C. (2009). M-Sec promotes membrane nanotube formation by interacting with Ral and the exocyst complex. Nat. Cell Biol..

[18] Kimura S., Hase K., Ohno H. (2012). Tunneling nanotubes: Emerging view of their molecular components and formation mechanisms. Exp. Cell Res..

[19] Lokar M., Iglič A., Veranič P. (2010). Protruding membrane nanotubes: Attachment of tubular protrusions to adjacent cells by several anchoring junctions. Protoplasma.

[20] He K.M., Luo W.X., Zhang Y.L., Liu F., Liu D., Xu L., Qin L., Xiong C., Lu Z., Fang X. (2010). Intercellular transportation of quantum dots mediated by membrane nanotubes. ACS Nano.

[21] Tosi G., Vilella A., Chhabra R., Schmeisser M.J., Boeckers T.M., Ruozi B., Vandelli M.A., Forni F., Zoli M., Grabrucker A.M. (2014). Insight on the fate of CNS-targeted nanoparticles. Part II: Intercellular neuronal cell-to-cell transport. J. Controlled Release.

[22] Abel M.P., Riese S.R., Schlicker O., Bukoreshtliev N.V., Gerdes H.H., Spatz J.P., Rustom A. (2011). Microstructured platforms to study nanotube-mediated long-distance cell-to-cell connections. Biointerphases.

[23] You R., Li X., Liu Y., Liu G., Lu S., Li M. (2014). Response of filopodia and lamellipodia to surface topography on micropatterned silk fibroin films. J. Biomed. Mater. Res..

[24] Bettinger C.J., Langer R., Borenstein J.T. (2009). Engineering substrate topography at the micro- and nanoscale to control cell function. Angew. Chem. Int. Ed..

[25] Nikkhah M., Edalat F., Manoucheri S., Khademhosseini A. (2012). Engineering microscale topographies to control the cell substrate interface. Biomaterials.

[26] Matschegewski C., Staehlke S., Loeffler R., Lange R., Chai F., Kern D.P., Beck U., Nebe B.J. (2010). Cell architecture-cell function dependencies on titanium arrays with regular geometry. Biomaterials.

[27] Kang K., Choi S.-E., Jang H.S., Cho W.K., Nam Y., Choi I.S., Lee J.S. (2012). *In vitro* developmental acceleration of hippocampal neurons on nanostructures of self-assembled silica beads in filopodium-size ranges. Angew. Chem. Int. Ed..

[28] Austefjord M.W., Gerdes H.H., Wang X. (2014). Tunneling nanotubes: Diversity in morphology and structure. Commun. Integr. Biol..

[29] Vepari C., Kaplan D.L. (2007). Silk as a biomaterial. Prog. Polym. Sci..

[30] Kundu B., Rajkhowa R., Kundu S.C., Wang X. (2012). Silk fibroin biomaterials for tissue regenerations. Adv. Drug Deliv. Rev..

[31] Zhang Q., Yan S., Li M. (2009). Silk fibroin based porous materials. Materials.

[32] Lawrence B.D., Pan Z., Liu A., Kaplan D.L., Rosenblatt M.I. (2012). Human corneal limbal epithelial cell response to varying silk film geometric topography *in vitro*. Acta Biomater..

[33] Koch K., Bhushan B., Jung Y.C., Barthlott W. (2009). Fabrication of artificial Lotus leaves and significance of hierarchical structure for superhydrophobicity and low adhesion. Soft Matter.

[34] Lou E., Fujisawa S., Morozov A., Barlas A., Romin Y., Dogan Y., Gholami S., Moreira A.L., Manova-Todorova K., Moore M.A.S. (2012). Tunneling nanotubes provide a unique conduit for intercellular transfer of cellular contents in human malignant pleural mesothelioma. PLoS One.

[35] Gerdes H.H., Carvalho R.N. (2008). Intercellular transfer mediated by tunneling nanotubes. Curr. Opin. Cell Biol..

[36] Sherer N.M., Mothes W. (2008). Cytonemes and tunneling nanotubules in cell-cell communication and viral pathogenesis. Trends Cell Biol..

[37] Marzo L., Gousset K., Zurzolo C. (2012). Multifaceted roles of tunneling nanotubes in intercellular communication. Front Physiol..

[38] Wittig D., Wang X., Walter C., Gerdes H.H., Funk R.H.W., Roehlecke C. (2012). Multi-level communication of human retinal pigment epithelial cells via tunneling nanotubes. PLoS One.

